# Flexibly designable wettability gradient for passive control of fluid motion via physical surface modification

**DOI:** 10.1038/s41598-023-33737-4

**Published:** 2023-04-20

**Authors:** Keita Funayama, Atsushi Miura, Hiroya Tanaka

**Affiliations:** Toyota Central R&D Labs., Inc., Nagakute, Aichi 480-1192 Japan

**Keywords:** Applied physics, Physical chemistry, Nanoscience and technology, Engineering

## Abstract

Modified solid surfaces exhibit unique wetting behavior, such as hydrophobicity and hydrophilicity. Such behavior can passively control the fluid flow. In this study, we experimentally demonstrated a wettability-designable cell array consisting of unetched and physically etched surfaces by reactive ion etching on a silicon substrate. The etching process induced a significant surface roughness on the silicon surface. Thus, the unetched and etched surfaces have different wettabilities. By adjusting the ratio between the unetched and etched surface areas, we designed one- and two-dimensional wettability gradients for the fluid channel. Consequently, fine-tuned channels passively realized unidirectional and curved fluid motions. The design of a wettability gradient is crucial for practical and portable systems with integrated fluid channels.

## Introduction

Microfluid channels have been investigated for chemical and biological applications such as high-sensitivity and portable sensors^1–6^. The active control of fluid motion in microchannels is a crucial technique to determine their performance^7–13^. To date, micromechanical valves^14,15^, pneumatic-controlled valves^16^, and electrically switchable surface modification^17–20^ have been explored. Such active control requires complicated fabrication procedures and external energy sources to achieve excellent controllability and reformation of wettability by external stimuli. Passive control of the fluid is another important approach^21–31^. This methodology enables us to control wettability using simple structures without reformable functions. As platforms of the passive control, aligned anisotropic structures (e.g., arrays of Janus pillars^32^ and concave curvature edge structures^33^) and different materials-based wettability gradient^34^ have been investigated. However, such approaches limit the flexibility of the channel design.

Because the etching process, e.g., reactive ion etching (RIE), modifies the surface state of a solid material^35,36^, unetched and etched surfaces exhibit different wettability properties, even when using a single material. The manufacture of microscopic unetched or etched surfaces using a semiconductor process enables flexible design and simple fabrication of functional surfaces with special wettability.

In this study, we experimentally investigated unidirectional and curved fluid channels with square-wettability-designable cell arrays. The unit cells consisted of an unetched area and an etched area with RIE. The RIE processes created nanoscopic recesses on the silicon surface, thereby providing different wettabilities on the unetched surfaces. The wettability of the silicon surfaces was tuned by adjusting the ratio of the unetched and etched surface areas. The fine-tuned etching patterns of the individual cells passively created one- and two-dimensional (1D and 2D) wettability gradients on the silicon substrate. It enabled us to control the direction and shape of the fluid channels by flexibly designing the form of the wettability gradient. Our platform can contribute to the passive control of fluid motion in practical applications.

## Methods

### Wettability design of silicon surfaces

**Figure 1:**
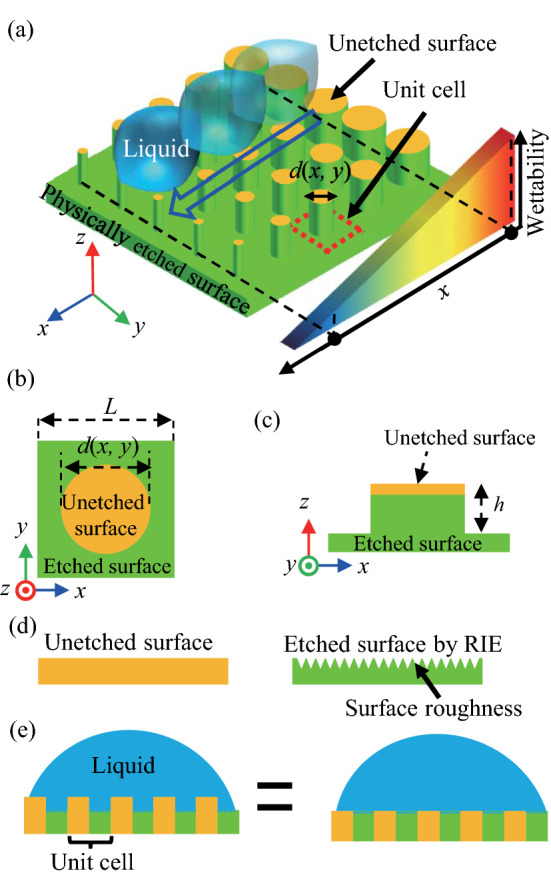
(**a**) Schematic of the surface comprising the wettability designable cells, which provide the 1D wettability gradient. A unit cell (enclosed by the red dotted square) has unetched (yellow) and physically etched (green) regions. The parameter $d\left(x, y\right)$ is the diameter of the pillar. (**b**) Top and (**c**) side view schematics of the unit cells. The side length of the unit cell and height of the pillar are $L=5 \mathrm{\mu m}$ and $h=0.2 \mathrm{\mu m}$, respectively, in our fabricated channels. (**d**) Schematics of cross-section for unetched and etched surfaces. (**e**) Schematics of cross-section for the surface paved with the unit cells (left schematic) and equivalent composite surface (right schematic).

Herein, we present a silicon-based wettability-designable fluid channel with the RIE process. Figure 1a shows the surface structure of the wettability- designable fluid channel. Our fluid channel was paved with square unit cells consisting of physically unetched and etched surfaces via RIE, as shown in Fig. 1b,c. The unit cell (enclosed by the broken red square in Fig. 1a) had unetched and etched areas. The etching process formed a depressed (green) region. The unetched (yellow) region remained as a cylindrical pillar at the center of the unit cell.

The wettability on the fluid channel was designed with two procedures. First, we prepared two surfaces with different wettabilities, i.e., the unetched and etched surfaces. The RIE process induces the roughness on the silicon surface, as shown in Fig. 1d. Owing to the roughness, the effective surface area increases. The hydrophilic wettability of such surfaces is expressed by Wenzel's law:

1 $$\mathrm{cos}\,{\theta }_{2}=r\,\mathrm{cos}\,{\theta }_{1}$$

where ${\theta }_{1 \left(2\right)}$ is the contact angle on the unetched (etched) surface, and $r$ is a roughness factor^37^. Secondly, we finely tuned the effective wettability on the unit cells by adjusting the fractional areas of the unetched (${r}_{1}$) and etched (${r}_{2}$) regions. The values of ${r}_{1}$ and ${r}_{2}$ varied by adjusting the pillar diameter, $d\left(x,y\right)$, which is described as:

2a $${r}_{1}=\frac{\pi {\left(d\left(x,y\right)/2\right)}^{2}}{{L}^{2}+\pi hd\left(x,y\right)},$$

2b $${r}_{2}=\frac{{L}^{2}-\pi {\left(d\left(x,y\right)/2\right)}^{2}+\pi hd\left(x,y\right)}{{L}^{2}+\pi hd\left(x, y\right)},$$

where $L$ and $h$ are the lengths of both sides of the unit cell and pillar height, respectively. When the two surfaces consist of hydrophilic materials, the water contacts the whole surface on the unit cells having surface asperities via the cylindrical pillars, as shown in the left schematic in Fig. 1e. This state is equivalent to the composite surface consisting of the two regions, which have different wettabilities, as shown in the right schematic in Fig. 1e. The effective contact angle $\theta$ on such a surface is described by Cassie’s equation^38^:

3 $$\mathrm{cos}\,\theta ={r}_{1}\mathrm{cos}{\theta }_{1}+{r}_{2}\mathrm{cos}{\theta }_{2},$$

Using Wenzel’s and Cassie’s laws, we modeled the unit cells with fine designability for their wettability.

Interestingly, the cell array created an anisotropic wettability gradient across the entire surface. Dropped liquid spreads and propagates toward the region with high hydrophilicity on such a gradient surface. For example, the fluid channel had a 1D wettability gradient, where $d\left(x,y\right)$ gradually decreased along the $x$- axis, whereas it remained constant along the $y$-axis, as shown in Fig. 1a. Such a 1D gradient provided a unidirectional fluid channel, that is, the liquid was likely to flow toward the positive direction along the $x$-axis, as shown by the blue arrow in Fig. 1a. Moreover, when $d\left(x,y\right)$ was varied along the $x$- and $y$-axes, the surface exhibited a 2D wettability gradient. Accordingly, the direction and shape of fluid channels can be flexibly designed. For example, a 2D wettability gradient provided a curved fluid channel.

### Fluid channel fabrication

The silicon substrate was cleaned by immersing it in a piranha solution (mixed concentrated H_2_SO_4_ and H_2_O_2_ at a ratio of 3:1), Milli-Q water (18 $\mathrm{M\Omega }{\mathrm{ cm}}^{-1}$), and dilute HF. The procedure of substrate cleaning eliminates organic impurities and the natural oxide layer on the substrate. We coated the substrate with hexamethyldisilazane and an electron beam (EB) resist (Zeon Corporation, ZEP520). The periodically aligned pillars were patterned by EB lithography (JEOL, JBX-6300FS). For surface modification, the silicon surfaces were physically etched by the RIE (SAMCO, RIE-10NR) process with Ar and CF_4_ gases at an etching power of 200 W and pressure of 2.0 Pa. Because of the high-power etching and low pressure of the reactive gases, the RIE process physically etched and induced surface roughness on the silicon surface. Finally, the substrate was again cleaned by the procedure of substrate cleaning to eliminate unwanted chemical hydrophilic groups that formed via the RIE process.

To elucidate the wettability of cell arrays, we prepared surfaces paved with (i) homogeneous cells for several constant values of pillar diameter, $d\left(x,y\right)={d}_{\mathrm{const}}$. In addition, we studied the surfaces paved with (ii) 1D and (iii) 2D inhomogeneous cells with 1D and 2D wettability gradients, respectively. We fabricated the unit cells with a side length of $L=5 \mathrm{\mu m}$ and a pillar height of $h=0.2 \mathrm{\mu m}$ in all surfaces in this study.

The surface (i) consisted of homogeneous 1000 × 1000 cells (array size: 5 × 5 mm^2^). The pillar diameter ${d}_{\mathrm{const}}$ was varied according to ${r}_{2}=$ 1.0, 0.93, 0.74, 0.54, 0.37, and 0. The conditions of ${r}_{2}=0$ and 1.0 respectively represented completely unetched and etched silicon surfaces. The value of ${r}_{2}$ was calculated using Eq. (2b). Wettability was evaluated by measuring the effective contact angles on the cell arrays by dropping 2 $\mathrm{\mu L}$ of water.

Surface (ii) comprised 1080 $\times$ 1980 cells (array size: 5.4 $\times$ 9.9 mm^2^). To form the 1D wettability gradient, we gradually changed $d\left(x, y\right)$ from 4.75 to 0.3 $\mathrm{\mu m}$ in steps of 0.05 $\mathrm{\mu m}$ per 22 cells (110 $\mathrm{\mu m}$) along the $x$-axis. On the $y$-axis, 1080 cells with the same values of $d\left(x, y\right)$ were periodically aligned in each row, i.e., $d\left(x, y\right)$ solely depends on $x$. Thus, the expression of $d\left(x, y\right)$ is described as:

4 $$d\left(x, y\right)=4.75-\left[\frac{x}{110}\right]\times 0.05,$$

where, $\left[\cdot \right]$ is Gauss symbol. Subsequently, the fluid channel had a wettability gradient along the $x$-axis and was flat on the $y$-axis. We repeatedly dropped 1 $\mathrm{\mu L}$ of water from the edge of the array. The passive motion of the dropped fluid was observed by taking a picture after each drop.

Surface (iii) was designed to have a 2D wettability gradient. We formed 1080 $\times$ 1980 cells (array size: 5.4 $\times$ 9.9 mm^2^) on the silicon surface. The pillar diameter $d\left(x, y\right)$ varied along both $x$ and $y$ axes. The maximum value of $d\left(x, y\right)$ was 4.75 $\mathrm{\mu m}$ at the center of above edge in the channel, $\left(x, y\right)=\left(0, w/2\right)$, where $w=5.4 \mathrm{mm}$ was the channel width along the $y$-axis. Along the $y$-axis, $d\left(x, y\right)$ increased (decreased) at a step of 0.05 $\mathrm{\mu m}$ per 27 cells (135 $\mathrm{\mu m}$) in $0\le y\le w/2$ ($w/2<y\le w$). The diameter $d\left(x, y\right)$ decreased at a step of 0.05 $\mathrm{\mu m}$ per 28 cells (140 $\mathrm{\mu m}$) along the $x$-axis. Thus, the expression of $d\left(x, y\right)$ is described as:

5 $$d\left(x, y\right)=\left\{\begin{array}{c}3.80+0.05\times \left[\frac{y}{135}\right]-0.05\times \left[\frac{x}{140}\right] if 0\le y\le \frac{w}{2}\\ 4.75-0.05\times \left[\frac{y-\frac{w}{2}}{135}\right]-0.05\times \left[\frac{x}{140}\right] if \frac{w}{2}<y\le w\end{array}\right..$$

The behavior of the fluid was examined by dropping water into the channel. Note that the unit for coordinate $x$ and $y$ is micrometer.

### Measurement setup

The conditions of the modified silicon surface were examined using X-ray photoelectron spectroscopy (XPS) (ULVAC PHI Inc., Quantera SXM), atomic force microscopy (AFM) (Hitachi High-Tech Science Corp., E-sweep), and transmission electron microscopy (TEM) (JEOL Ltd., JEM-ARM200F). We recorded the wetting behavior of water droplets on the silicon surface using a contact angle meter and analysis software (Kyowa Interface Science Co., Ltd., DMo-501, and FAMAS).

## Results and discussion

### Surface modification of silicon substrate by the RIE process

**Figure 2:**
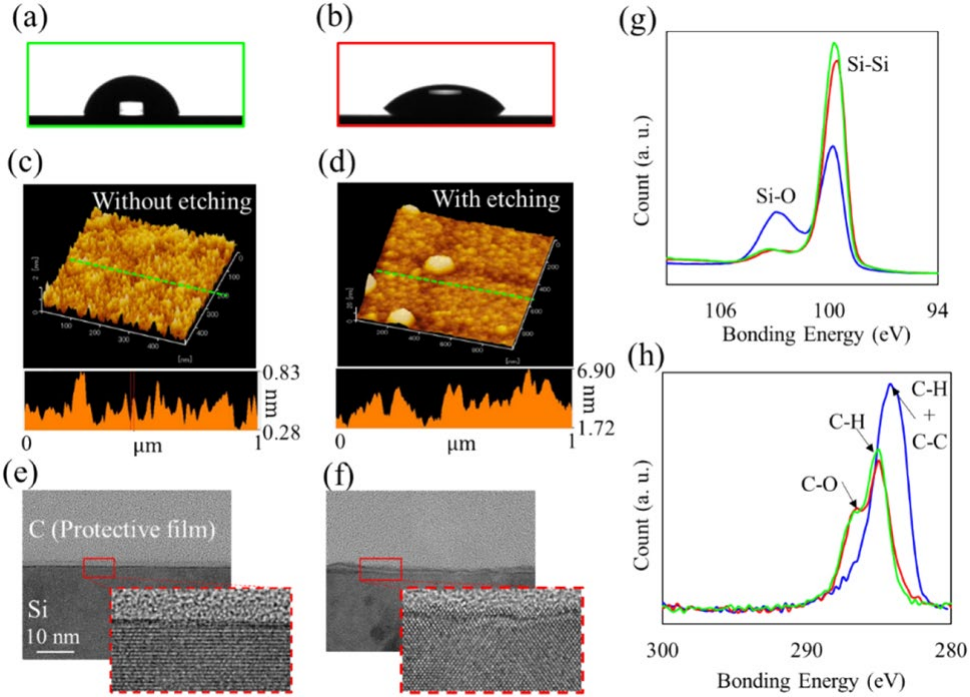
Side views of the water droplet on the cleaned silicon surface (**a**) without and (**b**) with plasma etching processes. Surface profiles of the silicon surface (**c**) without and (**d**) with the etching via AFM. The TEM images of the cross section of the silicon surfaces (**e**) without and (**f**) with etching. (**g**) Si2p and (**h**) C1s XPS spectra. Red, green, and blue lines represent process (A) (w/ RIE and w/ cleaning), (B) (w/o RIE and w/ cleaning), and (C) (w/ RIE and w/o cleaning), respectively.

The unetched (left schematic in Fig. 1d) and etched (right schematic in Fig. 1d) silicon surfaces exhibited different wettabilities. To observe this, we measured the wetting behavior on silicon surfaces with and without the RIE process. Figure 2a,b show the side views of the droplet when 2 $\mathrm{\mu L}$ of water were dropped onto the unetched and etched silicon surfaces. The contact angles were 80.3° (Fig. 2a) and 42.1^o^ (Fig. 2b) for the surfaces without and with the RIE process, respectively. The RIE process resulted in a more wettable surface on the silicon substrate. Note that both substrates were cleaned using the substrate cleaning procedure to eliminate unwanted chemical hydrophilic groups before the observation.

Such a discrepancy in wettability between the unetched and etched silicon surfaces is explained by the surface roughness, as presented in the literatures^39–42^. We evaluated the surface roughness of the unetched and etched silicon substrates using AFM. Figure 2c,d show the surface and height profiles, respectively, along the green dotted line. The etched silicon had a considerably rougher surface than the unetched silicon. The height difference for the surface without etching was approximately 0.55 nm. In contrast, the surface subjected to the etching process had a height difference greater than 5 nm. The rough surface was created through the RIE process because the etching ions (accelerated Ar ions, $\sim$ 0.1 nm) formed nanoscale recesses ($\sim$ sub-nanometers) on the surface. We note that the pillar’s surface roughness does not significantly contribute to wettability on the unit cells. In fact, the roughness factor via pillars in unit cells was $1<r<1.12$. In contrast, $r$ is 1 for the unetched surfaces and 4.73 for the etched surfaces with the nanoscale recesses, which were calculated from the AFM images.

Furthermore, we can visually capture the roughness differences between the unetched and etched silicon surfaces. Figure 2e,f show the TEM images of the cross-section of the unetched and etched silicon surfaces, respectively. As shown in Fig. 2e, the surface atoms had a crystalline arrangement. A flat profile was observed on the unetched surface. Meanwhile, in Fig. 2f, the flatness of the etched surface deteriorated owing to the distortion of the atomic layers. This result indicated an increase in the roughness of the silicon surface owing to the RIE process.

Chemical composition also changes the wettability^43–45^. However, in our approach, chemical wettability was a negligible effect because the functional groups were removed by the procedure of substrate cleaning with piranha solution, dilute HF, and pure water, which is denoted as *cleaning* in the following discussions. To clarify the contribution of the chemical compositions, we examined the XPS spectra for the silicon surface with (A) w/ RIE and w/ cleaning, (B) w/o RIE and w/ cleaning, (C) w/ RIE and w/o cleaning processes.

Figure 2g,h show the Si2p and C1s XPS spectra, respectively. The red line is the spectrum for the surface of process (A) (w/RIE and w/cleaning). The binding energies for the Si–Si, C–H, and C–O bonds were 99.75, 285, and 286.5 eV, respectively. The C–H and C–O bonds were associated with deposited organic matter from the atmosphere after the cleaning process. A negligible peak at 103.25 eV (Si–O bond) stemmed from the natural oxide film formed during the cleaning process and XPS measurements. The results suggested that there were no functional groups, such as silicon-oxygen or silicon-hydrogen compounds, on the surface for process (A). The spectra for process (B) are similar to those for process (A). Thus, the surfaces of processes (A) and (B) had similar morphologies in terms of chemical composition.

In contrast, the spectra for process (C) (w/RIE and w/o cleaning, represented by the blue line) were explicitly different from those for process (A). Specifically, we observed significant peaks for the Si–O and C–C bonds in process (C). The peaks of the C–H and C–C bonds imply the formation of hydrophilic groups on the silicon surface, thereby resulting in the Si–O bond. The etching gases (Ar and CF_4_) do not form an oxide layer, that is, silicon dioxide. Thus, the cleaning process can eliminate the chemical effects of plasma etching. Notably, the hydrophilic groups on the silicon surface provided a considerably different wettability from that of the cleaned surface. The measured contact angle was 6.7° (see Supporting Information for more details).

### Designability of wetting behavior for the etched silicon surface via RIE

**Figure 3:**
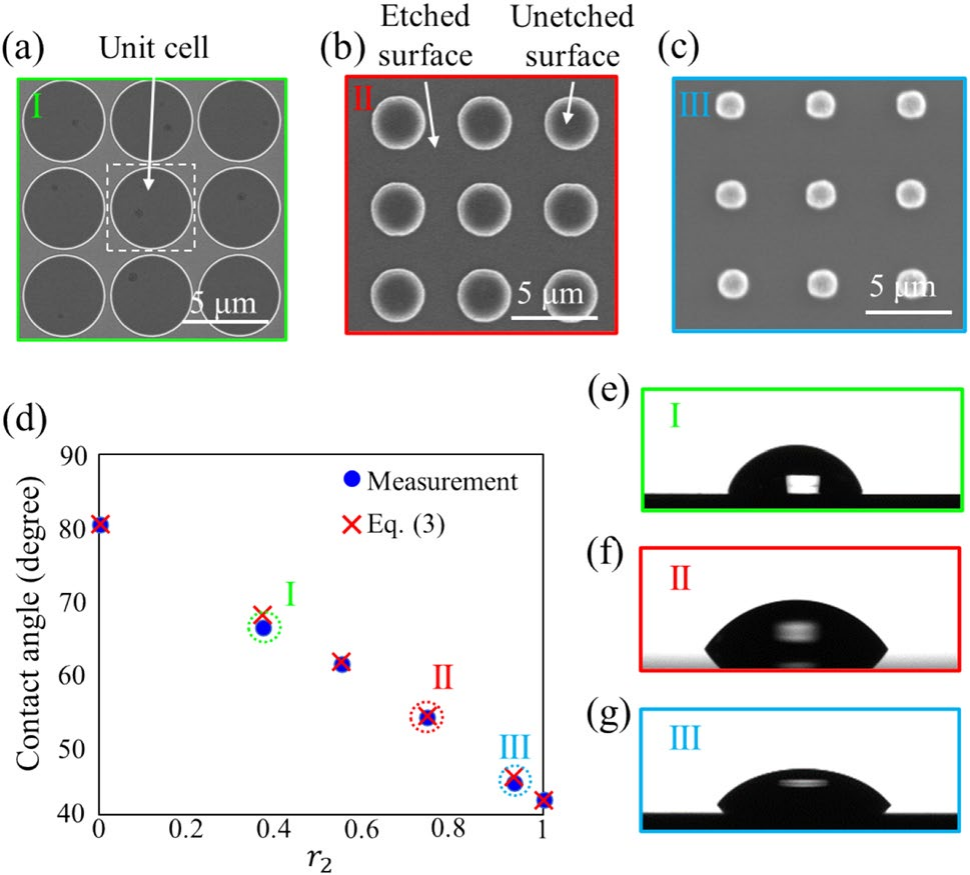
The SEM images of the surface consisting of the homogenous cell array with *r*_2_ = (**a**) $0.37$ (${d}_{\mathrm{const}}=$ 4.75 $\mathrm{\mu m}$), (**b**) 0.74 (${d}_{\mathrm{const}}=$ 3.0 $\mathrm{\mu m}$), and (**c**) 0.93 (${d}_{\mathrm{const}}=$ 1.5 $\mathrm{\mu m}$). (**d**) The measured and calculated contact angles as the function of ${r}_{2}$. The conditions of ${r}_{2}=0$ and 1 imply unetched and etched surfaces overall, respectively. The side-view photographs of the water droplet with *r*_2_ = (**e**) 0.37, (**f**) 0.74, and (**g**) 0.93. The contact angle and ratio ${r}_{2}$ in (**d**) were calculated from Eqs. (2b) and (3) with each designed value of ${d}_{\mathrm{const}}$, respectively.

Herein, we investigated the controllable range of the contact angle $\theta$ for the surface paved with homogeneous cells, surface (i). Figure 3a–c show SEM images of the fabricated cell array for ${r}_{2}=$ 0.37, 0.54, 0.74, and 0.93. The interior of the circle (i.e., the top of the pillar) was the unetched surface, and the other surface in the cell was etched. Figure 3d shows the contact angles as a function of the ratio of the etched area ${r}_{2}$. The measured contact angles (blue circle) decreased with increasing ${r}_{2}$, that is, the wettability on the silicon surface became more hydrophilic owing to the enlargement of the etched area. The experimental results agreed well with the contact angles calculated using Eq. (3) (Red crosses).

Figure 3e–g are the photographs of the water droplet for ${r}_{2}$ labeled as I, II, and III in Fig. 3a–c, respectively. The dome-shaped drop of water indicates that the periodically etched structure allows for fine-tuning of the wettability on the silicon surface.

### Wetting behavior of surface with inhomogeneous cell array

**Figure 4:**
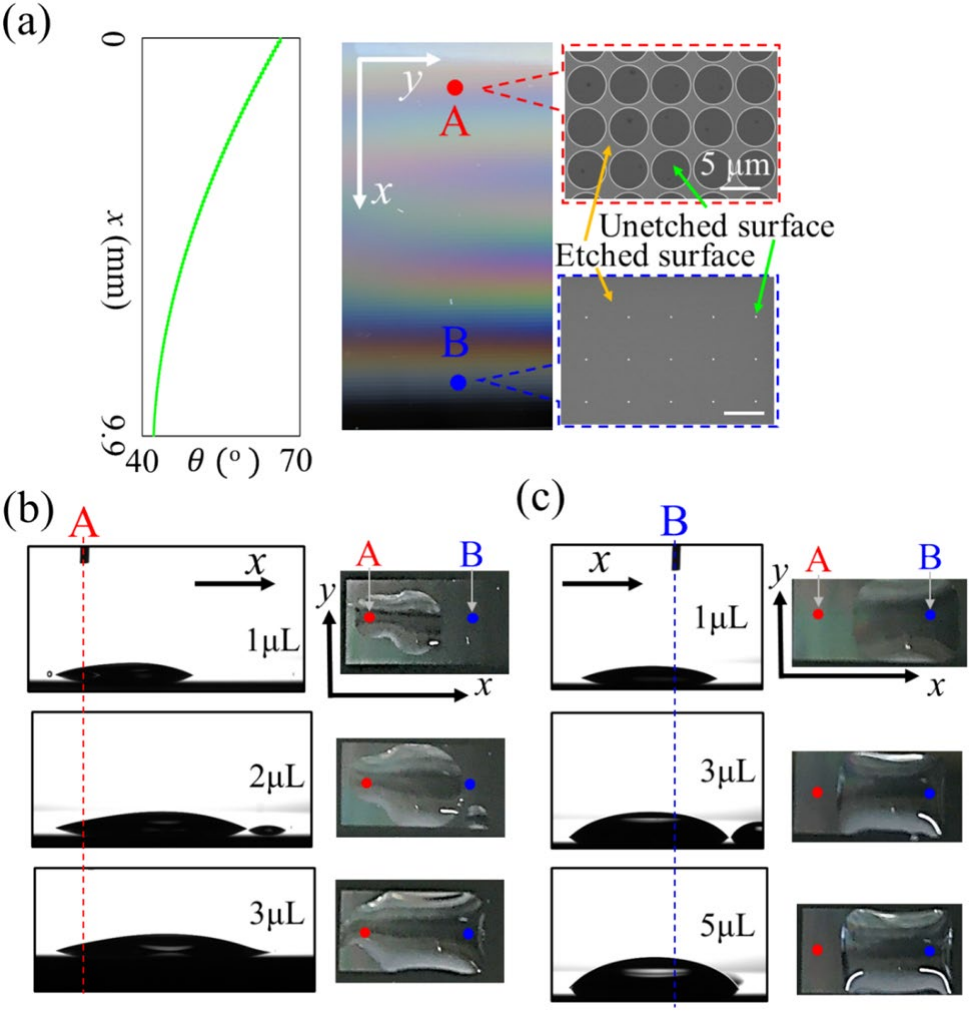
(**a**) Top-view photograph of the unidirectional fluid channel (center panel), SEM images around point A and B (right two panels), and the calculated contact angle on the $x$-axis (left panel). Side- and top-view photographs when repetitively dropping water from (**b**) point A and (**c**) B.

By individually designing $d\left(x, y\right)$ of the unit cells, we can create a wettability gradient on the silicon surfaces. First, we demonstrated the 1D wettability gradient on surface (ii), which provided a unidirectional fluid channel. Figure 4a shows a top view of the fabricated channel. The plot on the left in Fig. 4a shows the calculated contact angle along the $x$-axis. Surface (ii) has a monotonic slope of the contact angle corresponding to the shape of the wettability gradient. The right panels in Fig. 4a show SEM images at points A and B. At point A, the diameter of the unetched region $d\left(x, y\right)$ is significant. Thus, the surface has low wettability. In contrast, $d\left(x, y\right)$ was set to a negligible value to achieve high wettability near point B. Consequently, this surface structure monotonically decreased the wettability gradient along $x$-axis as shown in Fig. 1a.

By repetitively dropping 1 $\mathrm{\mu L}$ of water from point A and B, the motion of the water could be passively controlled. Figure 4b,c show the side and top views of the water droplet after each dropping step at points A and B, respectively. The broken lines represent the positions of the drop points A and B in the side views of Fig. 4b,c, respectively. When a droplet of water is dropped into a channel, it spreads according to the relationship between the contact angle and the self-weight of a droplet with a finite volume. In Fig. 4b, the droplet only spread toward point B as water was repetitively injected into the channel from point A. Finally, the droplet reached point B after dropping 3 $\mathrm{\mu L}$ of water. In contrast, in Fig. 4c, the left edge of the droplet was pinned when water was dropped from point B. The droplet remained around point B without spreading toward point A, even after dropping 5 $\mathrm{\mu L}$ of water. Therefore, the surface (ii) functioned passively as a unidirectional fluid channel.

**Figure 5:**
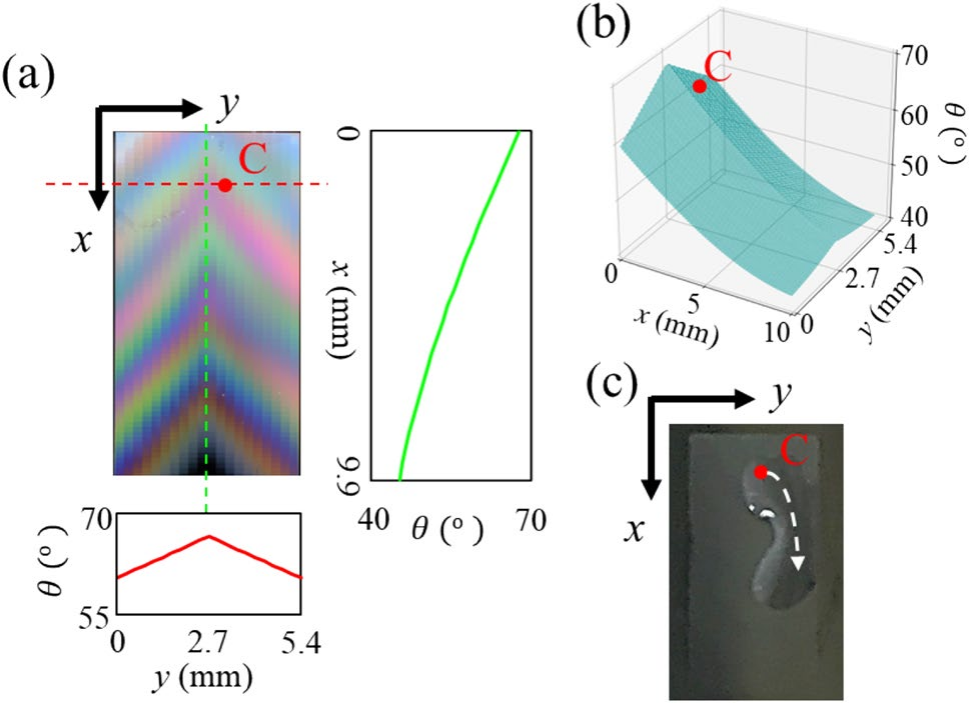
(**a**) Top view photograph of the fabricated fluid channel with 2D wettability gradient. Left and bottom graphs are the calculated contact angles in $x$- and $y$-axes. (**b**) 3D plot of the contact angle on the wettability gradient. (**c**) Top-view photograph when dropping the water from point C.

Furthermore, we demonstrated a curved fluid channel with a 2D wettability gradient. Figure 5a shows a top-view photograph of the channel with the slope of the wettability along $x$- and $y$-axes of surface (iii). The right (bottom) panel in Fig. 5a shows the contact angle calculated using Eq. (3) in the vertical green (horizontal red) dashed line in the photograph. The slopes of the contact angle indicated that surface (iii) had monotonic and concaved-down wettability along $x$- and $y$-axes, respectively. Figure 5b shows the 3D plot of the contact angle as a function of $x$ and $y$ on entire surface in the channel. In this channel, the wettability gradient was described as a combination of the two gradients along $x$- and $y$-axes. Along $x$-axis, the wettability gradient flows water from upper to lower regions in the channel. Along $y$-axis, the form of wettability gradient is convex shape where the contact angle has maximum and minimum values at the center and both side edges of the channel, respectively. The effective flow direction in the channel is expressed as a summation of the flow directions along $x$- and $y$-axes. Considering the gradient directions from Fig. 5b, the water dropped on point C is expected to flow toward lower-right in the channel. Figure 5c shows the behavior of the water dropping from point C. The dropped water moved toward the bottom- right edge of the channel, as depicted by the white arrow. The direction of the water fluid is in good agreement with the above argument.

## Conclusion

In conclusion, we experimentally investigated a wettability-designable cell array on a silicon surface. The designable range of the contact angle was ${80.3}^{^\circ }\le \theta \le {42.1}^{^\circ }$, as determined by the etching pattern with RIE. The fine-tuned distribution of the unetched and etched regions allowed 1D and 2D wettability gradients. Thus, the cell arrays enabled the flexible design of the direction and shape of the fluid channels. Our wettability-designable cell array is a crucial platform to passively control the fluid motion (Supplementary Information S1).

## Supplementary Information

Supplementary Information.

## Data Availability

The data that support the findings of this study are available from the corresponding author upon reasonable request.
