# Supplementary material for: Flexibly designable wettability gradient for passive control of fluid motion via physical surface modification
Source: Sci Rep. 2023 Apr 20;13:6440. doi: 10.1038/s41598-023-33737-4 (PMC10119291; doi:10.1038/s41598-023-33737-4)
Supplement: Supplementary file 1 — Supplementary Information. [file 41598_2023_33737_MOESM1_ESM.docx]

Supplementary Material for

"Flexibly designable wettability gradient for passive control of fluid motion via physical surface modification"

Keita Funayama^*^, Atsushi Miura, Hiroya Tanaka

Toyota Central Research & Development Laboratories, Inc., Nagakute 480-1192, Japan

E-mail: [funayama@mosk.tytlabs.co.jp](mailto:funayama@mosk.tytlabs.co.jp)


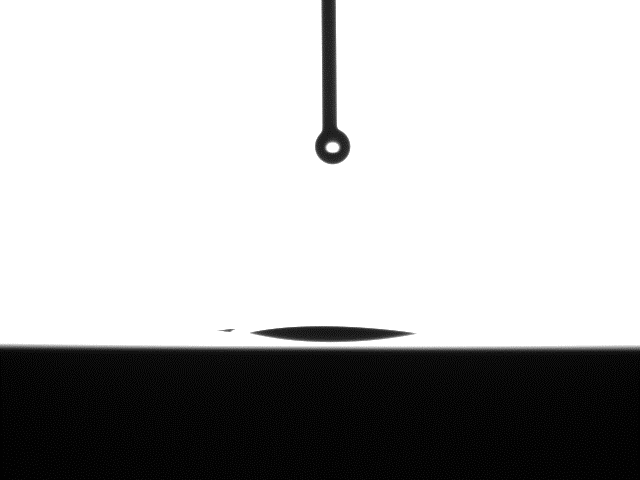


Fig. S1 Side view of the water droplet on the plasma-etched silicon substrate without cleaning. The measured contact angle was 6.7^o^. As the results of the surface analyses in main manuscript, we found the high wettability coming from the hydrophilic groups formed by the etching process.
